# Community screening for dementia in Kenya: Lessons learnt from the DEM‐SKY program

**DOI:** 10.1002/alz.086152

**Published:** 2025-01-09

**Authors:** Christine W. Musyimi, David Ndetei, Levi A. Muyela, Elizabeth Kasimu Mutunga, Joe Masila, Nicolas Farina

**Affiliations:** ^1^ Africa Mental Health Research and Training Foundation, Nairobi Kenya; ^2^ University of Nairobi, Nairobi, Nairobi Kenya; ^3^ Africa Mental Health Research and Training Foundation, Nairobi, Nairobi Kenya; ^4^ Alzheimer’s and Dementia Organization Kenya, Nairobi Kenya; ^5^ Makueni County referral Hospital, Makueni Kenya; ^6^ University of Plymouth, Plymouth United Kingdom

## Abstract

**Background:**

The Integration and Evaluation of a Community‐Level **Dem**entia **S**creening Programme in rural **K**en**Y**a (DEM‐SKY) program, supported by the Davos Alzheimer’s Collaborative, leverages existing community resources to promote early detection of dementia among older adults and strengthen health system policy and practice in Kenya.

**Methods:**

The DEM‐SKY program consisted of two key components: a training component and dementia screening component using then brief Community Screening Instrument for Dementia (brief‐CSID). The training component was divided into two stakeholder groups: Community Health Workers (CHWs) and hospital staff. Each group had different training needs to ensure the successful implementation of DEM‐SKY. Over a six‐month period, ten CHWs approached 3,546 older adults in Makueni County aged 60+ years to offer dementia screening. We adopted a process evaluation framework and used qualitative interviews to help identify factors that hampered or facilitated the successful adoption of dementia screening within the rural Kenyan context.

**Results:**

Our findings revealed that 18% of older adults in rural Kenya have cognitive and functional impairment indicative of dementia, translating to over half a million older adults in Kenya. The DEM‐SKY program also illustrated the importance of collaborative partnerships with policy makers, health care providers and community members while encouraging a proactive approach in tackling challenges right from project conception. Through emerging themes and experiences, we also highlight opportunities that strengthened health care systems and promoted infrastructure readiness to address dementia care gaps during implementation of a novel dementia screening program in a low‐resource setting. These include use of existing community resources, community‐based participatory approaches and stigma‐reduction activities prior to program implementation.

**Conclusion:**

The implementation of the DEM‐SKY program has been a landmark in early detection of dementia in Kenya. It forms the first community‐based dementia detection program implemented in rural Kenya with limited specialists and inadequate infrastructure, hence providing timely initiation of care cascade for individuals with cognitive and functional impairment. This program could be used to inform governments to drive innovations that promote early detection and healthy ageing; and invest in future development and implementation of treatment and support systems for older adults with dementia in low‐resource settings.

